# Supplementary material for: Suppression of gut colonization by multidrug-resistant Escherichia coli clinical isolates through cooperative niche exclusion
Source: Nat Commun. 2025 Jul 1;16:5426. doi: 10.1038/s41467-025-61327-7 (PMC12215308; doi:10.1038/s41467-025-61327-7)
Supplement: Supplementary file 1 — Supplementary Information [file 41467_2025_61327_MOESM1_ESM.pdf]

Supplementary Fig. 1

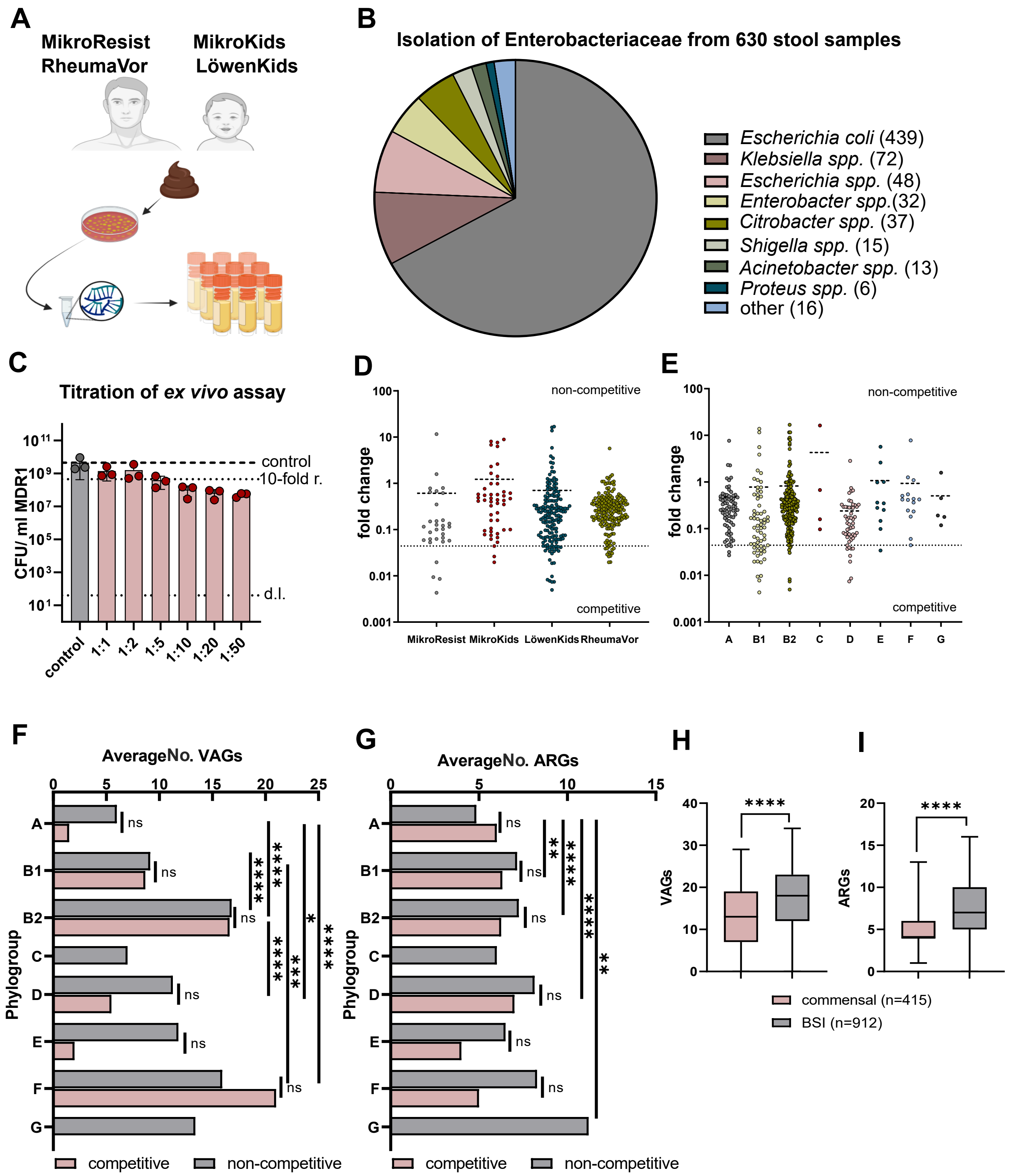

Supplementary Figure 1: Commensal *E. coli* strains show different competitive effects. (A) Schematic overview of experimental workflow. Fecal samples of healthy donors were plated on selective MacConkey agar plates. Isolated colonies were identified using 16S-colony PCR and cryo-preserved. Created with biorender. (B) Pie chart of the bacterial composition of the strain collection on genus level. (C) Fold change of co-cultures of MR102 and MDR1 in different ratios. (D) Fold change of co-cultures to control of all strains (n=430) split in cohorts. (E) Fold change of co-cultures to control of all strains (n=430) split in phylogroups. (F) Average no. of virulence genes in each phylogroup. (G) Average no. of AMR genes in each phylogroup. (H) Comparison of virulence genes of commensal strains to a reference collection of clinical isolates (BSI). (I) Comparison of AMR genes of commensal strains to a reference collection of clinical isolates (BSI). P values indicated represent a RM Two-Way Anova p<0.05, **p<0.01, ***p<0.001, ****p<0.0001

Supplementary Fig. 2

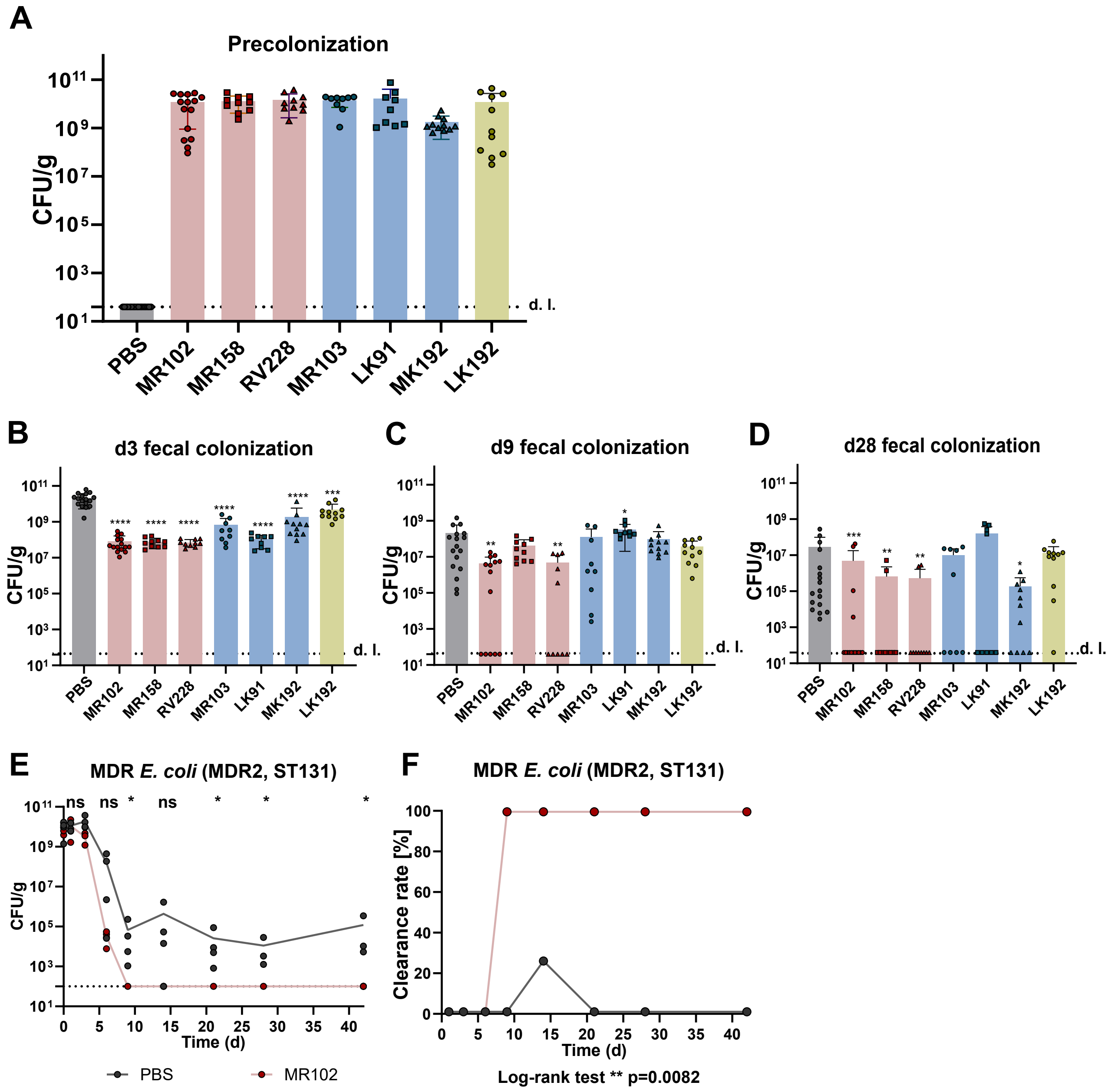

Supplementary Figure 2: Specific commensal *E. coli* strains enable decolonization of MDR *E. coli* in a preventive and therapeutic manner. (A) Precolonization levels of different commensal *E. coli* strains. (B-D) CFU/g of *E. coli* MDR1 of single mice at various time points of colonization. Geometric mean and SEM of two to three independent experiments with n=9-17 mice per group. P values indicate a nonparametric Kruskal-Wallis test $p<0.05$, $^{**}p<0.01$, $^{***}p<0.001$, $^{****}p<0.0001$. (E) Resulting fecal colonization levels of *E. coli* MDR2 after different time points of colonization. Geometric mean and SEM of one experiments with n= 4 mice per group. P-values represent the Log-rank (Mantel-Cox) test with $^{*}p < 0.05$. (F) Clearance kinetics of *E. coli* MDR2 after different time points of colonization (clearance = CFU/g below the detection limit in feces). P-values represent the Log-rank (Mantel-Cox) test with $^{**}p < 0.001$.

Supplementary Fig. 3

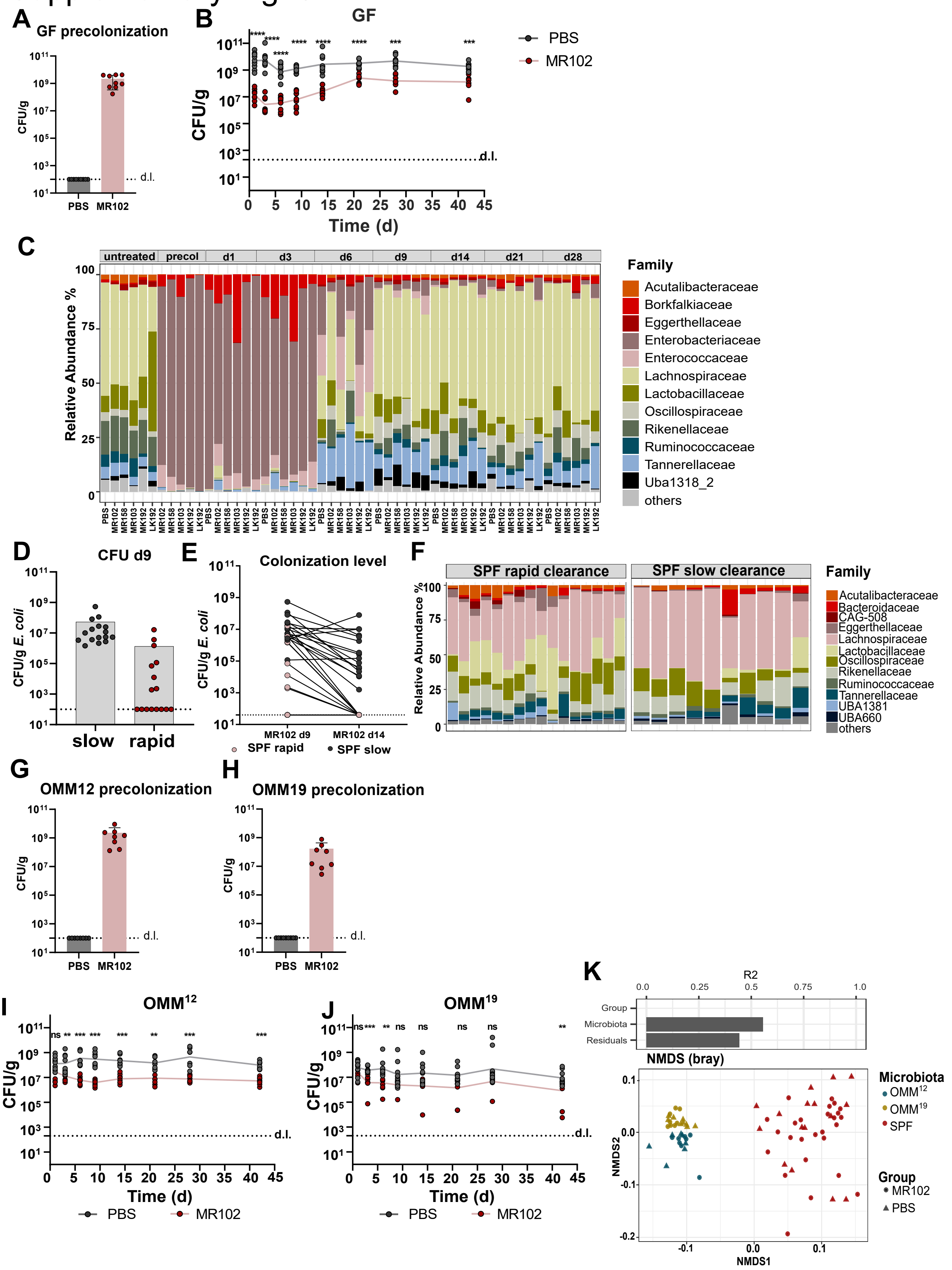

Supplementary Figure 3: The protective effect is dependent on the microbial context. (A) The resulting fecal burden of *E. coli* MR102 after precolonization in GF mice. (B) Resulting fecal burden of *E. coli* MDR1/ MR102 after different time points of colonization. Geometric mean and SEM of two independent experiments with n=8-9 mice per group. P values indicate a nonparametric Kruskal-Wallis test p<0.05, **p<0.01, ***p<0.001, ****p<0.0001. (C) Average microbiome composition at different time points throughout the experiment on the family level. (D,E) CFU/g at day 9 and 14 of slow and rapid clearance phenotype. (F) Microbiome composition at day nine of slow and rapid clearance phenotype mice on the family level. (G, H) The resulting fecal burden of *E. coli* MR102 after precolonization in OMM mice. (I) Resulting fecal burden of *E. coli* MDR1/ MR102 after different time points of colonization. Geometric mean and SEM of two independent experiments with n=8-9 mice per group. P values indicate a nonparametric Kruskal-Wallis test p<0.05, **p<0.01, ***p<0.001, ****p<0.0001. (K) Β-diversity of fecal samples of mice at day nine (data from two to seven independent experiments, n=7-33 mice per group) based on functionality was analyzed using the Bray-Curtis dissimilarity matrix and NMDS.

Supplementary Fig. 4

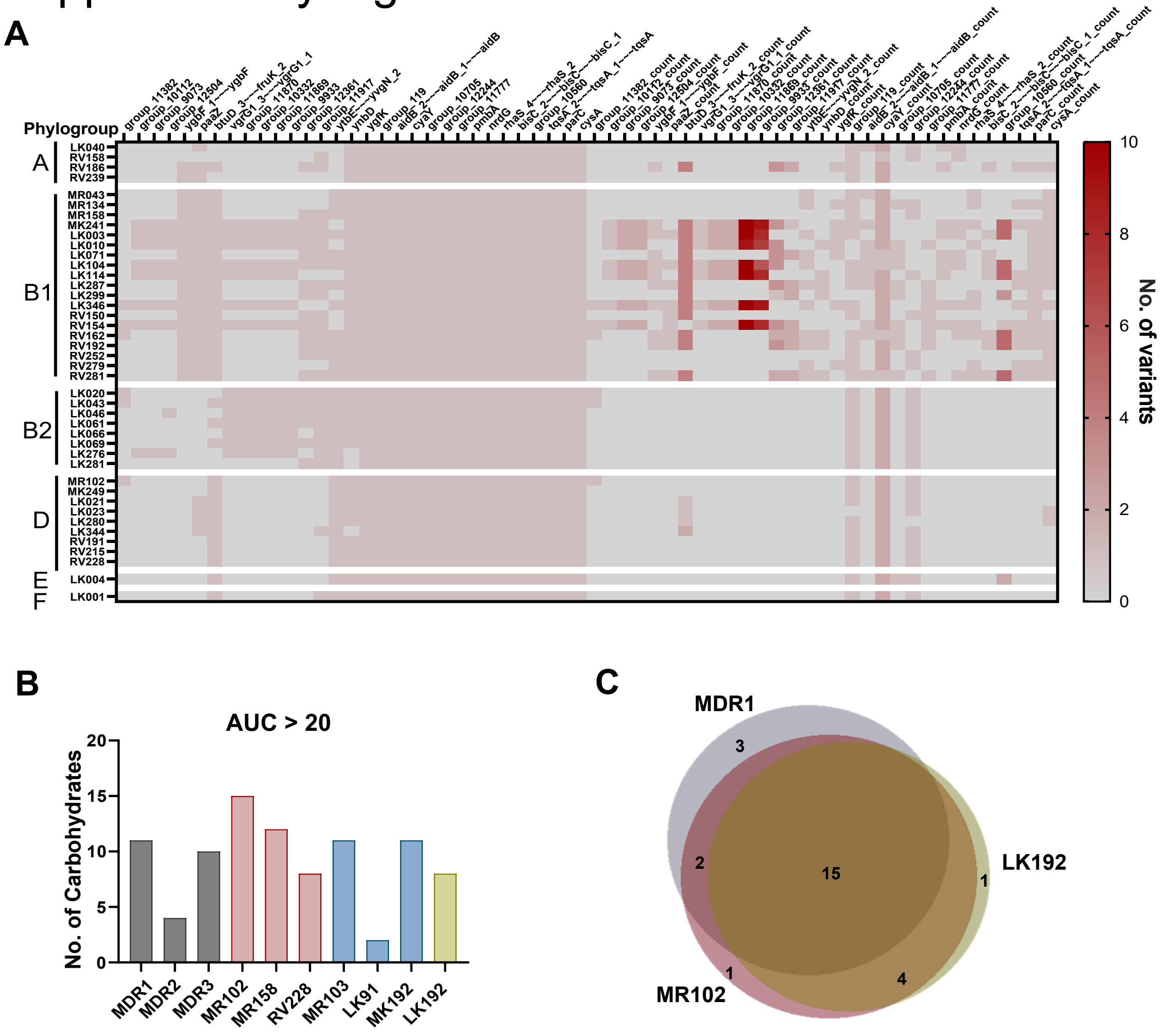

Supplementary Figure 4: Distinct carbohydrate utilization is involved in intra-species competition. (A) Heatmap showing variants of identified unitigs associated with competitiveness in competitive strains. (B) Bar plot showing the number of carbohydrates with an AUC>20 for each strain. (C) Venn diagrams displaying *E. coli* MDR1, MR102, and LK192 carbon source overlap.

Supplementary Fig. 5

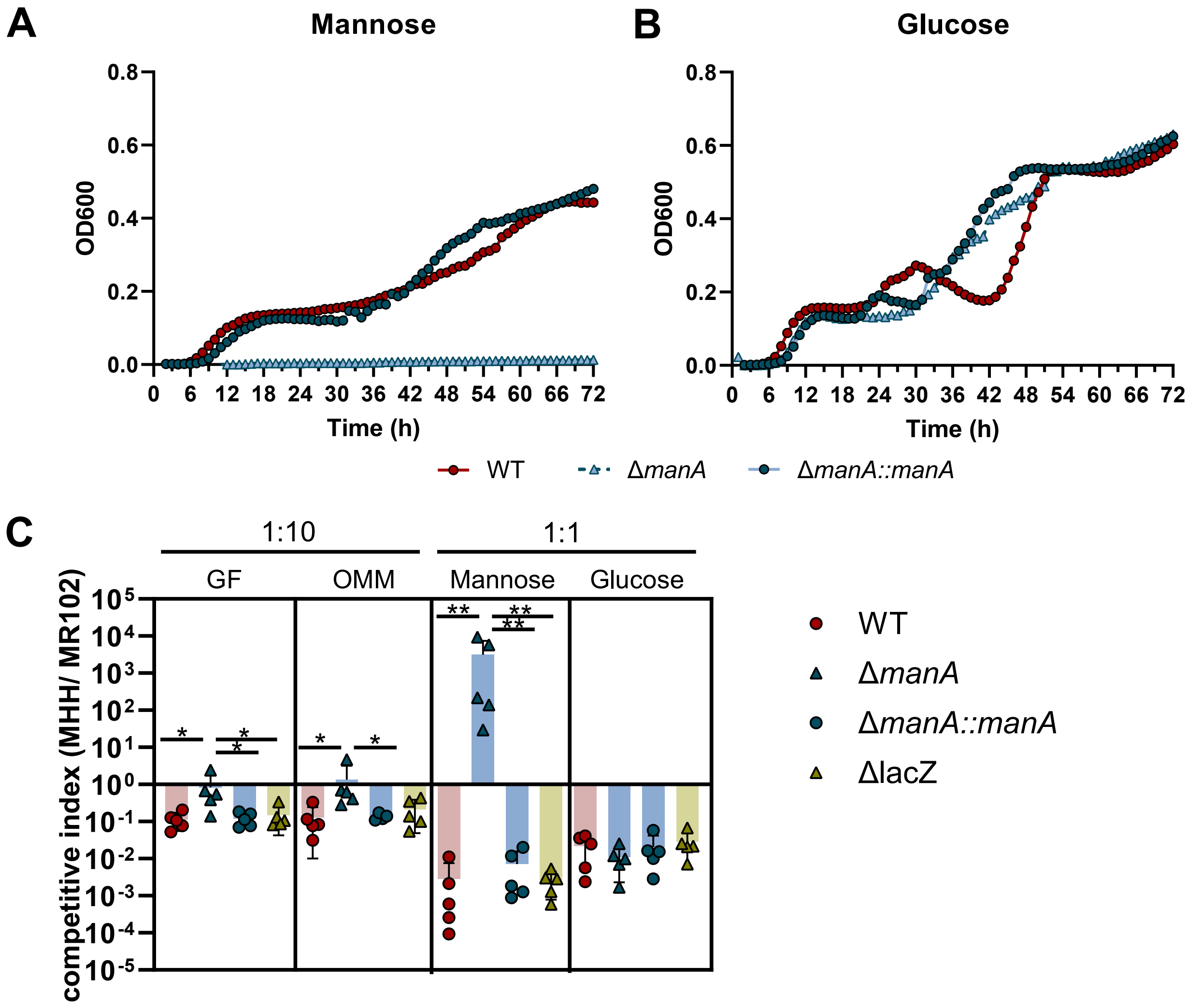

Supplementary Figure 5: Protective *E. coli* strain is superior to MDR *E. coli* in direct competition for specific carbohydrates. Growth curves of *E. coli* MR102 WT, ΔmanA, and ΔmanA::manA in MM9 media supplemented with 5 g/L (A) mannose and (B) glucose. Growth at 37 °C was monitored by hourly measurements of the OD600 over 72 h. Data represents the mean of triplicates. (C) Results of competition assay in cecum content of GF and OMM mice and MM9 supplemented with 5 g/L of respective carbon source. Mean and SEM of five independent experiments performed in duplicates. P values indicated represent a nonparametric Kruskal-Wallis test *p<0.05.

Supplementary Fig. 6

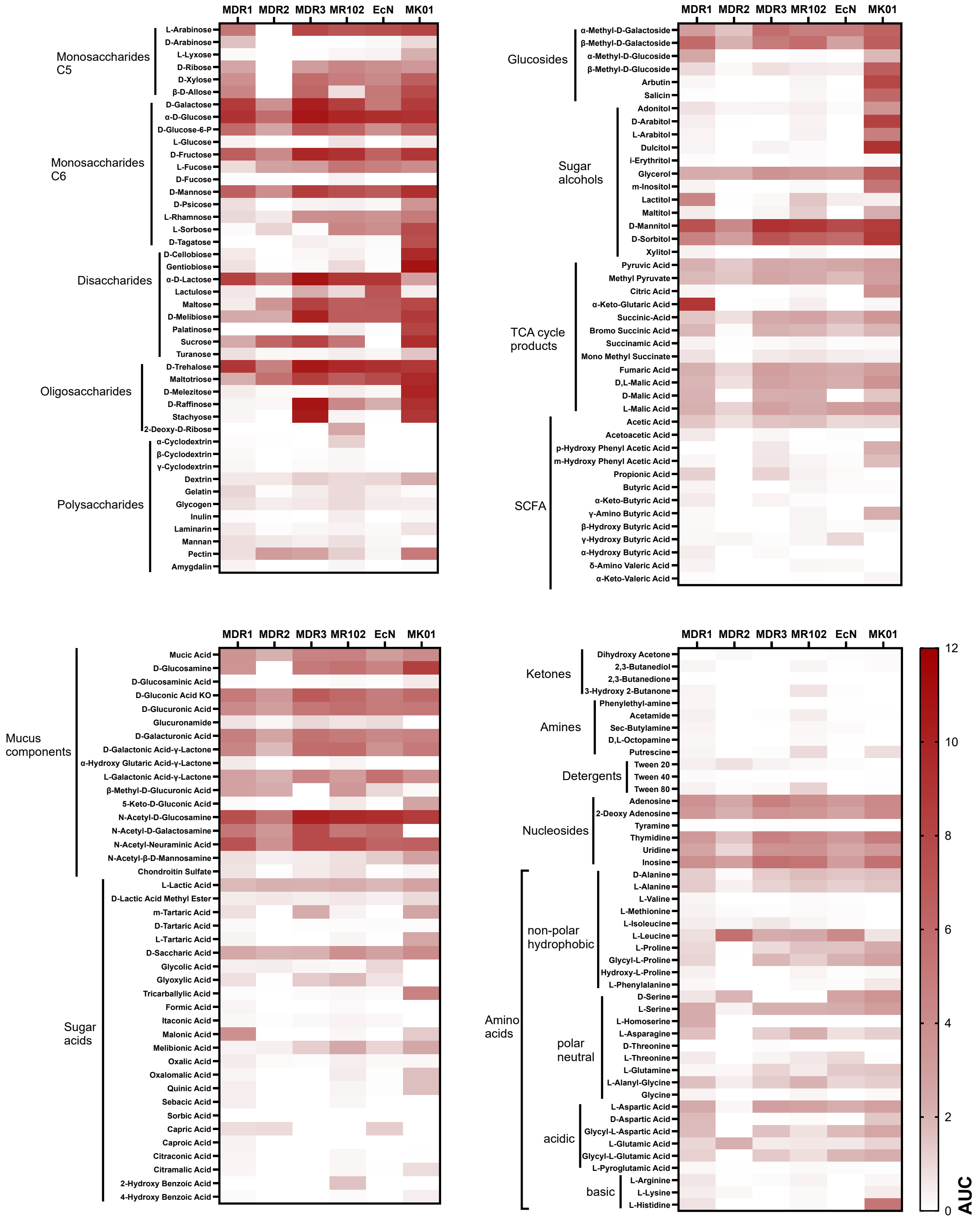

Supplementary Figure 6: A combination of metabolically diverse Enterobacteriaceae can enlarge the target spectrum of MDR-E. Heatmap showing AUC of 24 h of growth curves of *E. coli* and *K. oxytoca* strains in Biolog® plates. Results of two to three independent experiments.

Supplementary Fig. 7

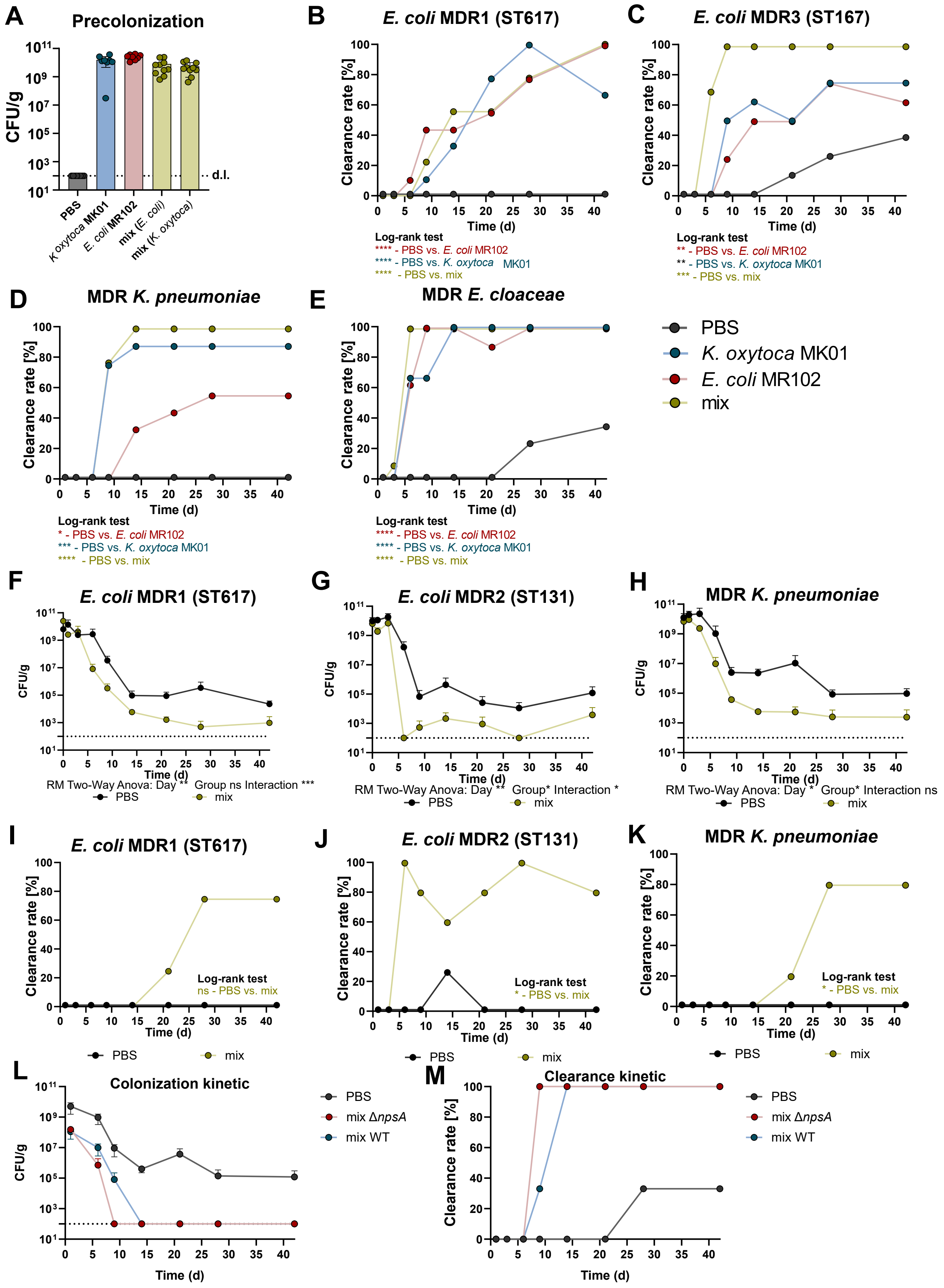

Supplementary Figure 7: A combination of metabolically diverse Enterobacteriaceae can enlarge the target spectrum of MDR-E. (A) Precolonization levels in CFU/g of *K. oxytoca* MK01 and *E. coli* MR102. (B-E) Clearance kinetics of MDR-E at different time points of colonization (clearance = CFU/g below the detection limit in feces). P-values represent the Log-rank (Mantel-Cox) test with $^*p < 0.05$, $^{**}p < 0.01$, $^{***}p < 0.001$, $^{****}p < 0.0001$. (F-H) Resulting fecal burden of MDR-E after different time points of colonization. Mean and SEM of two independent experiments with n=3-5 mice per group. (I-K) Clearance kinetics of MDR-E at different time points of colonization (clearance = CFU/g below the detection limit in feces). P-values represent the Log-rank (Mantel-Cox) test with $^*p < 0.05$. (L) Resulting fecal colonization levels of *E. coli* MDR1 after different time points of colonization. Data represents the mean and SEM of one experiment with n=3-5 mice per group. (M) Clearance kinetics of *E. coli* MDR1 (clearance = CFU/g below the detection limit in feces).

Supplementary Table 1

	MikroResist	MikroKids	RheumaVor	LöwenKids	Total
**Donors**	**59**	**54**	**253**	**264**	**630**
**Isolated bacterial species**					
Escherichia coli	34	52	187	166	439
Escherichia marmotae	1	-	-	4	5
Escherichia fergusonii	13	2	11	17	43
Enterobacter hormaechei	2	2	1	2	7
Enterobacter ludwigii	1	-	2	3	6
Enterobacter cloaceae	-	-	2	7	9
Enterobacter tabaci	-	-	1	-	1
Enterobacter asburiae	-	-	-	1	1
Enterobacter sp.	-	-	5	3	8
Shigella flexneri	2	-	-	-	2
Shigella sonnei	2	2	2	7	13
Citrobacter farmeri	-	2	2	1	5
Citrobacter freundii	-	4	8	10	22
Citrobacter braakii	-	-	2	1	3
Citrobacter koseri	-	-	2	-	2
Citrobacter portucalensis	-	-	1	1	2
Citrobacter murlinae	-	-	-	1	1
Citrobacter sp.	1	-	-	1	2
Klebsiella oxytoca	8	21	6	6	41
Klebsiella pneumoniae	11	3	8	1	23
Klebsiella variicola	1	-	-	2	3
Klebsiella grimontii	-	-	-	2	2
Klebsiella michiganensis	-	-	-	1	1
Klebsiella quasipneumoniae	1	-	-	1	2
Pantoea agglomerans	1	3	-	2	6
Proteus mirabilis	1	-	-	2	3
Proteus vulgarus	-	-	1	1	2
Proteus sp.	1	-	-	-	1
Hafnia avei	-	1	3	2	6
Hafnia paralvei	-	1	1	-	2
Hafnia sp.	1	-	1	-	2
Acinetobacter baumanii	1	-	1	8	10
Acinetobacter jonsonii	-	-	-	1	1
Acinetobacter sp.	2	-	-	-	2

Supplementary Table 1: Isolated bacterial species in this study.

Supplementary Table 2

Strain label	Source	MDR	ST	Phylogroup	WGS
E. coli 2365332 (MDR1)	Hannover Medical School	NDM-1, Oxa-48	ST617	A	yes
E. coli NRZ-51853 (MDR16)	National Reference Centre for Gram-negative Nosocomial Pathogens	NDM-5	ST410	C	yes
E. coli NRZ-53221 (MDR13)	National Reference Centre for Gram-negative Nosocomial Pathogens	NDM-5	ST46	A	yes
E. coli NRZ-54519 (MDR9)	National Reference Centre for Gram-negative Nosocomial Pathogens	NDM-5	ST405	D	yes
E. coli NRZ-54875 (MDR14)	National Reference Centre for Gram-negative Nosocomial Pathogens	NDM-5	ST617	A	yes
E. coli NRZ-55476 (MDR10)	National Reference Centre for Gram-negative Nosocomial Pathogens	NDM-5	ST1284	A	yes
E. coli NRZ-55652 (MDR3)	National Reference Centre for Gram-negative Nosocomial Pathogens	NDM-5	ST167	A	yes
E. coli NRZ-55838 (MDR8)	National Reference Centre for Gram-negative Nosocomial Pathogens	NDM-5	ST38	D	yes
E. coli NRZ-56035 (MDR11)	National Reference Centre for Gram-negative Nosocomial Pathogens	NDM-5	ST1702	A	yes
E. coli NRZ-56231 (MDR7)	National Reference Centre for Gram-negative Nosocomial Pathogens	NDM-5	ST156	B1	yes
E. coli NRZ-56236 (MDR5)	National Reference Centre for Gram-negative Nosocomial Pathogens	NDM-5	ST940	B1	yes
E. coli NRZ-56589 (MDR6)	National Reference Centre for Gram-negative Nosocomial Pathogens	NDM-5	ST648	F	yes
E. coli NRZ-56922 (MDR4)	National Reference Centre for Gram-negative Nosocomial Pathogens	NDM-5, OXA-244	ST361	A	yes
E. coli NRZ-21236 (MDR2)	National Reference Centre for Gram-negative Nosocomial Pathogens	loss of porin, ESBL	ST131	B2	yes
E. coli NRZ-26260 (MDR12)	National Reference Centre for Gram-negative Nosocomial Pathogens	OXA-48	ST38	D	yes
E. coli NRZ-44583 (MDR15)	National Reference Centre for Gram-negative Nosocomial Pathogens	IMP-14	ST2197	A	yes
E. coli PBIO729 (dog, MDR17)	Helmholtz Institute for One Health	blaCTX-M-15, blaTEM-1, blaOXA-1,tet(AR) aadA, aac(6')-ib-cr	ST131		yes
E. coli PBIO730 (crow, MDR18)	Helmholtz Institute for One Health	blaCTX-M-15, tet(AR), sul1,2, strAB, aadA, aac(3)-II, mph(A), mrx, mphR	ST648		yes
E. coli O127:H6 strain E2348/69 (EPEC)	Jochen Hühn, Helmholtz Centre for Infection Research	no			
E. cloaceae 2444764	Hannover Medical School	VIM			yes
P. mirabilis 1826728	Hannover Medical School	NDM			yes
S. enterica Serovar Typhimurium SL1344 strain EM12442	Marc Erhardt, Humboldt University Berlin	no			
K. oxytoca MK01	Helmholtz Centre for Infection Research (HZI)	no			yes
K. pneumoniae YA21621	University hospital Magdeburg	NDM-1	ST395		yes

Supplementary Table 2: Strains used in this study.

Supplementary Table 3

Phylogroup	Strains (n)	Competitive strains (n/ % of phylogroup)	Non-competitive strai (n/ % of phylogroup)
**A**	74	4/ 5.4%	70/ 94.6%
**B1**	65	19/ 29.2%	46/ 70.8%
**B2**	188	8/ 4.2%	180/ 95.7%
**C**	3	0/ 0%	3/ 100%
**D**	53	9/ 17%	44/ 83%
**E**	11	1/ 9.1%	10/ 90.9%
**F**	15	1/ 6.7%	14/ 93.3%
**G**	5	0/ 0%	5/ 100%

Supplementary Table 3: Distribution of commensal *E. coli* strains in different phylogroups.
