# Supplementary material for: Suppression of gut colonization by multidrug-resistant Escherichia coli clinical isolates through cooperative niche exclusion
Source: Nat Commun. 2025 Jul 1;16:5426. doi: 10.1038/s41467-025-61327-7 (PMC12215308; doi:10.1038/s41467-025-61327-7)
Supplement: Supplementary file 2 — Description of Additional Supplementary Files [file 41467_2025_61327_MOESM2_ESM.pdf]

Title: Supp. Data 1

Description: Genotypic and phenotypic characteristics of commensal E. coli isolates used in ex vivo competition assays.

Title: Supp. Data 2

Description: Presence of virulence-associated antimicrobial resistance genes in E. coli isolates.

Title: Supp. Data 3

Description: Presence and absence of unitigs identified with GWAS analysis in E. coli strains.
