# Supplementary material for: Suppression of gut colonization by multidrug-resistant Escherichia coli clinical isolates through cooperative niche exclusion
Source: Nat Commun. 2025 Jul 1;16:5426. doi: 10.1038/s41467-025-61327-7 (PMC12215308; doi:10.1038/s41467-025-61327-7)
Supplement: Supplementary file 6 — Reporting Summary [file 41467_2025_61327_MOESM6_ESM.pdf]

nature portfolio

Corresponding author(s): Till Strowig

Last updated by author(s): Jun 4, 2025

Reporting Summary

Nature Portfolio wishes to improve the reproducibility of the work that we publish. This form provides structure for consistency and transparency in reporting. For further information on Nature Portfolio policies, see our Editorial Policies and the Editorial Policy Checklist.

Statistics

For all statistical analyses, confirm that the following items are present in the figure legend, table legend, main text, or Methods section.

n/a	Confirmed	
☐	☒	The exact sample size (*n*) for each experimental group/condition, given as a discrete number and unit of measurement
☐	☒	A statement on whether measurements were taken from distinct samples or whether the same sample was measured repeatedly
☐	☒	The statistical test(s) used AND whether they are one- or two-sided<br>*Only common tests should be described solely by name; describe more complex techniques in the Methods section.*
☒	☐	A description of all covariates tested
☐	☒	A description of any assumptions or corrections, such as tests of normality and adjustment for multiple comparisons
☐	☒	A full description of the statistical parameters including central tendency (e.g. means) or other basic estimates (e.g. regression coefficient) AND variation (e.g. standard deviation) or associated estimates of uncertainty (e.g. confidence intervals)
☐	☒	For null hypothesis testing, the test statistic (e.g. *F*, *t*, *r*) with confidence intervals, effect sizes, degrees of freedom and *P* value noted<br>*Give P values as exact values whenever suitable.*
☒	☐	For Bayesian analysis, information on the choice of priors and Markov chain Monte Carlo settings
☒	☐	For hierarchical and complex designs, identification of the appropriate level for tests and full reporting of outcomes
☒	☐	Estimates of effect sizes (e.g. Cohen's *d*, Pearson's *r*), indicating how they were calculated

Our web collection on statistics for biologists contains articles on many of the points above.

Software and code

Policy information about availability of computer code

Data collection	The NGS data generated in this study have been deposited in the ENA (European Nucleotide Archive) database under accession code PRJEB76066 [https://www.ebi.ac.uk/ena/browser/view/PRJEB76066]. All other data generated in this study are provided in the Supplementary Information/Source Data file.
Data analysis	Usearch8.1 software package (16S rRNA gene analysis), GraphPad Prism v9.1 (Statistical Analysis)

For manuscripts utilizing custom algorithms or software that are central to the research but not yet described in published literature, software must be made available to editors and reviewers. We strongly encourage code deposition in a community repository (e.g. GitHub). See the Nature Portfolio guidelines for submitting code & software for further information.

Data

Policy information about availability of data

All manuscripts must include a data availability statement. This statement should provide the following information, where applicable:

- Accession codes, unique identifiers, or web links for publicly available datasets
- A description of any restrictions on data availability
- For clinical datasets or third party data, please ensure that the statement adheres to our policy

16S rRNA gene sequencing data have been deposited in the NCBI (Bioproject Database) under the accession number: PRJEB76066

Research involving human participants, their data, or biological material

Policy information about studies with human participants or human data. See also policy information about sex, gender (identity/presentation), and sexual orientation and race, ethnicity and racism.

Reporting on sex and gender	N/A
Reporting on race, ethnicity, or other socially relevant groupings	N/A
Population characteristics	N/A
Recruitment	N/A
Ethics oversight	N/A

Note that full information on the approval of the study protocol must also be provided in the manuscript.

Field-specific reporting

Please select the one below that is the best fit for your research. If you are not sure, read the appropriate sections before making your selection.

[x] Life sciences [ ] Behavioural & social sciences [ ] Ecological, evolutionary & environmental sciences

For a reference copy of the document with all sections, see nature.com/documents/nr-reporting-summary-flat.pdf

Life sciences study design

All studies must disclose on these points even when the disclosure is negative.

Sample size	Sample size for mouse studies was chosen according to institutional directives and in accordance with the 3Rs rules (Replacement, Reduction and Refinement) guiding principles underpinning the humane use of animals in research, but no statistical analyses were performed to predetermine the sample sizes. N=4 is the minimum number of independent subjects to be used for statistical analysis to be performed.
Data exclusions	No data were excluded from the study.
Replication	All attempts at replication were successful, with multiple mice in each group (see sample size above). All experiments were done at least twice, if feasible, each with multiple technical replicates
Randomization	Mice were randomly allocated to different treatments. We ensured that in each experiment all mice were siblings and shared the same cage for microbiome homogenization prior to the experiment.
Blinding	The investigators were not blinded to group allocation as blinding does not apply to this study because the investigators needed to identify the cages of mice for subsequent colonization resistance tests.

Reporting for specific materials, systems and methods

We require information from authors about some types of materials, experimental systems and methods used in many studies. Here, indicate whether each material, system or method listed is relevant to your study. If you are not sure if a list item applies to your research, read the appropriate section before selecting a response.

Materials & experimental systems

n/a	Involved in the study	
[x]	[ ]	Antibodies
[x]	[ ]	Eukaryotic cell lines
[x]	[ ]	Palaeontology and archaeology
[ ]	[x]	Animals and other organisms
[x]	[ ]	Clinical data
[x]	[ ]	Dual use research of concern
[x]	[ ]	Plants

Methods

n/a	Involved in the study	
[x]	[ ]	ChIP-seq
[x]	[ ]	Flow cytometry
[x]	[ ]	MRI-based neuroimaging

Animals and other research organisms

Policy information about studies involving animals; ARRIVE guidelines recommended for reporting animal research, and Sex and Gender in Research

Laboratory animals	C57BL/6N SPF mice were bred at the animal facilities of the Helmholtz Center for Infection Research (HZI) under enhanced specific pathogen-free (SPF) conditions. Germ-free C57BL/6NTac mice and OMM C57BL/6NTac mice were bred in isolators in the germ-free facility at the HZI. Animals used in experiments were gender and age matched. Female and male mice with an age of 8-12 weeks were used.
Wild animals	The study did not involve wild animals
Reporting on sex	For all animal experiments, groups were age- and gender-matched.
Field-collected samples	This study did not involve samples collected from the field.
Ethics oversight	The study was approved by the Lower Saxony State Office for Nature, Environment and Consumer Protection (LAVES), Oldenburg, Lower Saxony, Germany; permit No. 33.19-42502-04-21/3817.

Note that full information on the approval of the study protocol must also be provided in the manuscript.
